# Artisanal food of animal origin as reservoir of putative pathogenic *Escherichia coli*: a combined genomic and *in vivo* approach

**DOI:** 10.3389/fmicb.2025.1718380

**Published:** 2026-01-21

**Authors:** Frédérique Pasquali, Cecilia Crippa, Alex Lucchi, Gerardo Manfreda

**Affiliations:** Department of Agricultural and Food Sciences, Alma Mater Studiorum – University of Bologna, Bologna, Italy

**Keywords:** aEPEC/ExPEC hybrid, artisanal food, atypical enteropathogenic *Escherichia coli*, extraintestinal pathogenic *Escherichia coli*, galleria mellonella, source traceability, whole genome sequencing

## Abstract

The lack of a full automation and control of environmental parameters might result in potential risk of microbial contamination in small-scale production plants such as artisanal cheese and salami Italian productions. In a previous study, genomes of 33 *E. coli* isolates were sequenced. In the present study, the pathogenicity potential of *E. coli* strains was investigated by: (1) phylogenomic comparison with 202 public genomes of human, animal and environmental Italian origin; (2) pathogenicity assessment of strains with virulence patterns predicting specific *E. coli* pathotypes by using larvae of *Galleria mellonella* as *in vivo* infection model. Phylogenetic reconstruction revealed raw material and not the processing environment as source of salami contamination. Moreover, close proximity of some strains isolated from salami production with wild boar and extraintestinal human public strains was observed suggesting pigs and wild boar as potential reservoirs of pathogenic *E. coli*. The virulome of salami strains revealed the presence of genes already described as gene markers of atypical enteropathogenic *E. coli* (aEPEC; *bfp*-, *eae*+). Interestingly the analysis of virulence genes pointed toward additional genomes which showed genetic markers previously described as strongly associated to and/or extraintestinal pathogenic *E. coli* (ExPEC). *In vivo* experiments, confirmed the higher pathogenicity of strain 5STM5 with genetic pattern corresponding to hybrid aEPEC/ExPEC and two strains 3CP1522 and 6MB5 of cheese and salami production, respectively, with virulence genes previously associated to ExPEC pathotype. The combined approach pointed toward two genes *espC* for aEPEC, as well as *malX* for ExPEC which were significantly enriched in clinical genomes in comparison to genomes of other origins. These genes are worth of future investigations which could help to assess the risk for consumers after the consumption of contaminated artisanal food.

## 1 Introduction

In recent years, the demand for more genuine food has been shifting consumer choice toward artisanal foods (Almli et al., 2011; Cirne et al., 2019). Locally produced dairy and meat-based foods such as soft cheese and salami are among foods rising interest of consumers searching for food perceived as healthier (Roccato et al., 2017; Jaramillo-Bedoya et al., 2021). In particular in Italy, already since 2017, 76% of Italian consumers declared to be oriented to national products with specific focus on Protected Designation of Origin (PDO) and Protected Geographical Indication (PGI; Eurispes, 2017). Specifically in the dairy sector, at present 48% of Italy’s cow’s milk supply is dedicated to DOP/IGP cheeses (Assolatte, 2025). However, artisanal foods are produced in small-scale production plants, where the lack of a full automation and control of environmental parameters might result in potential risk of microbial contamination (Roccato et al., 2017; Jaramillo-Bedoya et al., 2021; Pasquali et al., 2023; Pasquali et al., 2022).

Escherichia coli is a facultative, anaerobic Gram-negative versatile bacterium. From harmless inhabitant to pathogenic colonizer of humans and animals, this bacterium has been extensively studied [Kaper et al., 2004; Anjum et al., 2021; ECDC, EFSA Panel on Biological Hazards (BIOHAZ), and EMA Committee for Medicinal Products for Veterinary Use (CVMP), 2017]. Within pathogenic *E. coli*, different pathotypes have been identified resulting in a spectrum of clinical syndromes ranging from mild, self-limited gastroenteritis to urinary tract infections and meningitis (Kaper et al., 2004). Within intestinal pathogenic *E. coli*, enteropathogenic *E. coli* (EPEC) have been reported. As enteric bacterial pathogen, EPEC has been described as a relevant factor associated to diarrhea in Humans of pediatric age in developing countries (Gomes et al., 2016; Ochoa and Contreras, 2011). Recently, EPEC has re-emerged associated with sporadic human cases or outbreaks in both developed and developing Countries (lee et al., 2022; Platenkamp and Mellies, 2018). Regarding outbreaks, several food categories were pinpointed as vehicles of transmission identifying specific EPEC strains as foodborne pathogens. Rocket salad was identified as source of contamination in 42 confirmed cases of EPEC infection in 2016 in Finland with symptoms ranging from none to diarrhea, vomiting and fever (Kinnula et al., 2018). In South Korea, 13 children in an elementary school were infected after consumption of water contaminated foods such as seasoned cucumber and chives (Lim et al., 2020). Besides plant-based foods, also food of animal origin can be contaminated by EPEC. EPEC was detected in more than 50% of eviscerated chicken carcasses in Argentina as well as in frozen carcasses in Iraq (Alonso et al., 2011; Taha and Yassin, 2019). Moreover, samples of raw dairy and meat products of bovine origin were positive for EPEC in Iran (Abri et al., 2019).

At present, EPEC are identified based on their pattern of virulence genes. Similar to enterohemorrhagic *Escherichia coli* (EHEC), all EPEC carry the *eae* gene, coding for intimin and included in the Locus for enterocyte effacement (LEE) associated to the characteristic attaching and effacing lesion (Kaper et al., 2004). Differently from EHEC, all EPEC do not carry the *stx1* and *stx2* genes coding for Shiga toxins. EPEC can be additionally divided in two groups namely “typical” and “atypical” EPEC based on the presence or absence of the *bfp* gene, respectively. The *bfp* gene is essential for biosynthesis of bundle-forming pili and it is located in the EPEC adhesion factor (EAF) plasmid (Kaper et al., 2004). Typical EPEC are considered diarrheagenic strains especially in children, whereas more controversial is the pathogenicity of atypical (a)-EPEC which have been isolated from both diseased and healthy Humans (Slinger et al., 2017; Ochoa and Contreras, 2011).

Along with intestinal pathogenic *E. coli*, pathotypes of extraintestinal pathogenic *E. coli* (ExPEC) have been reported as relevant Human pathogens associated with a variety of outcomes from meningitis in neonates to bacteremia in older adults and urinary tract infections in young healthy women (Russo and Johnson, 2003). In foods, ExPEC were isolated commonly in poultry meat, as well as in pork and beef although with a less extent. Poultry, pork and, with a minor extent, beef, have been associated to ExPEC lineages which emerged in the 1990s and they are still accounting for a large fraction of ExPEC diseases in humans (Manges and Johnson, 2012; Meena et al., 2023). ExPEC ST131 was detected in poultry and UTI isolates in Canada as well as in pork and UTI in Danmark and Norway (Vincent et al., 2010; Trobos et al., 2009). Direct link of human UTI lineage ExPEC ST69 to food, primarily pork and chicken meat, was suggested by PFGE similarities and pathogenicity assessment of food isolates in *in vivo* UTI mouse model (Vincent et al., 2010; Jakobsen et al., 2012). PFGE similarities were encountered between clinical and cow samples of ExPEC ST69 in US (Ramchandani et al., 2005). Multidrug-resistant ExPEC ST10 isolates were retrieved from human clinical samples, retail chicken and pork meat (although with limited PFGE similarity) in Canada (Bergeron et al., 2012). Regarding the molecular bases of ExPEC pathotype, several virulence factors were identified: (1) adhesins (i.e., *papACEFG*—P fimbriae), (2) iron acquisition systems (i.e., *iuc*—aerobactin), (3) protectins (i.e., *kpsM II—*kpsM II group 2 capsule), (4) invasins (i.e., *ibeABC*—cell invasion into the host tissue) and (5) toxins [i.e., *hylA*—*α*-haemolysin, cytotoxic necrotizing factor (*cnf*) and cytolethal distending toxin (*cdt*); Sora et al., 2021; Sarowska et al., 2019; Dale and Woodford, 2015; Onlen Guneri et al., 2022].

Although several virulence factors have been described in both aEPEC and ExPEC, the prediction of the final phenotypic outcome is challenging due to the high complexity of the virulome and the potential differential expression of its genes (Peirano et al., 2013). In this context, a particular relevance is covered by *in vivo* infection models essential to evaluate the pathogenic outcome of strains with different virulence patterns. Along with mouse and chicken animal models, other non-vertebrates animal models have been recently arising such as the larvae of the greater wax moth *Galleria mellonella* (Serrano et al., 2023; Rehman et al., 2017; Green et al., 2015; Antão et al., 2008; Ménard et al., 2021). In particular, this invertebrate has increasingly been used as an alternative model host in microbial pathogenesis studies. Adhering to the 3R principle of replacement, reduction and refinement, *Galleria mellonella*, as an invertebrate infection model, is not subject to Directive 2010/63/EU on the protection of animals used for scientific purposes. With its innate immune system very similar to mammal’s one, larvae of *G. mellonella* provides unprecedent opportunity for a first evaluation (or confirmation) of pathogenicity of bacterial strains. The translatability of results was documented by Velikova and colleagues, who observed that the pathogenicity in *G. mellonella* of different serotypes and mutants of *Streptococcus suis* was in agreement with pathogenicity observed in piglets (*Sus scrofa*; Velikova et al., 2016). Additional studies suggested the utility of this *in vivo* model to evaluate the pathogenic potential specifically of ExPEC and EPEC (Ménard et al., 2021; Williamson et al., 2014; Leuko and Raivio, 2012; Pasquali et al., 2025).

In a previous study, genomes of 33 *E. coli* isolates collected from Italian artisanal food productions were sequenced and their virulome and resistome characterized. Virulence gene patterns predicted putative pathogenicity for three genomes (Crippa et al., 2024). In the present study, the pathogenicity potential of previously sequenced *E. coli* was further investigated by: (1) phylogenomic comparison with 202 public genomes of human, animal and environmental origin; (2) pathogenicity assessment of strains with virulence patterns predicting specific *E. coli* pathotypes by using larvae of *Galleria mellonella* as *in vivo* infection model.

## 2 Materials and methods

### 2.1 Rationale for isolate selection

In a prior investigation, a total of 1,170 samples were collected between January 2020 and May 2021 from raw materials, semi-finished and finished products, as well as environmental surfaces, across six production cycles at two artisanal facilities producing soft cheese and organic salami in Italy (Pasquali et al., 2022; Pasquali et al., 2023). Biotyping analysis identified *Escherichia coli* in three soft cheese samples (one semi-finished and two finished products) and in 30 samples from the salami production line. The latter included raw materials, semi-finished products aged for 18 weeks, and surfaces and equipment such as tables and fillers located in the processing environment (Crippa et al., 2024).

### 2.2 Whole genome sequencing

In order to investigate the virulome of 33 *E. coli* isolates, the DNA was previously extracted using the MagAttract HMW DNA Kit (Qiagen, Milan, Italy) and sequenced on a Illumina NovaSeq platform (Illumina, Milan, Italy). Reads were assembled *de novo* using Unicycler v0.5.0 (Wick et al., 2017), and the assembled contigs were screened with ABRicate v1.0.1 (Seemann, 2020) using ResFinder (Bortolaia et al., 2020) and ecoli_VFdatabases (Crippa et al., 2024). In the present study, for comparison purposes to published papers, sequence types (STs) were assigned using mlst v2.23.0 (Seemann, 2022), based on the PubMLST database (Jolley and Maiden, 2010), whereas O: H serotypes and phylogroups were predicted *in silico* using the EnteroBase typing tools (Zhou et al., 2020).

### 2.3 SNP-based phylogenetic analysis

In order to infer phylogenetic relationships among *E. coli* strains isolated from artisanal food of animal origin and *E. coli* strains from other sources (human, environment, animal) SNP calling was performed incorporating both the 33 Italian *E. coli* strains and 202 publicly available *E. coli* genomes sourced from Enterobase (Dyer et al., 2025). Public genomes were selected based on Country (Italy), Collection year: 2020–2023, and their metadata including *s*erotypes, STs and lineages were retrieved from the Database (Supplementary Table S1). SNP identification and tree construction were, respectively, carried out with Snippy v.4.6.0 (Seemann, 2015) and PhyML v.3.1 (Guindon et al., 2010), using the reference genome *Escherichia coli* str. K-12 substr. MG1655 (NCBI RefSeq assembly no. GCF_000005845.2). The latter was implemented to build a maximum likelihood phylogenetic tree based on an alignment of core SNPs produced with snippy-core. The tree was visualized and annotated with metadata using iTOL v6 (Letunic and Bork, 2024). Pairwise SNP distances were calculated with snp-dists v0.6.3 (Taouk et al., 2025).

### 2.4 Virulome

To assess genetic determinants of virulence, all 235 assembled contigs were screened with ABRicate v1.0.1 (Seemann, 2020) using ecoli_vf database (Chen et al., 2016), setting a minimum coverage threshold of 90% and identity 60%. The presence/absence heatmap of virulence-associated genes identified in newly sequenced *E. coli* was visualized using the R package *pheatmap* v1.0.13. Among all genes detected by the database, only those typically described for ExPEC (Sarowska et al., 2019; Dale and Woodford, 2015) and EPEC (Bugarel et al., 2011; Perna et al., 2001; Garmendia et al., 2005; Karmali et al., 2003; Konczy et al., 2008) pathotypes were included in the heatmap. Moreover, samples were annotated by source type and sample origin.

To explore source-related differences in virulence genes among *Escherichia coli* isolates, statistical analysis was performed using R v4.5.0. Specifically, the prevalences of genes *malX* and *cnf1*, specifically found in genomes of putative EXPEC strains showing higher pathogenicity in *G. mellonella*, were compared between isolates of human and other (food, environment and animal) origin. The expected counts were calculated using the default method implemented in R’s chisq.test () function, based on marginal totals under the assumption of independence. Pearson’s chi-square test (χ2) was applied for *malX* as all expected cell counts were ≥ 5, whereas Fisher’s exact test was used for *cnf1* due to the presence of expected cell counts < 5. Odds ratios (ORs) with 95% confidence intervals (CIs) were calculated to quantify the strength of associations. Statistical significance was set at *α* = 0.05, without corrections for multiple testing.

### 2.5 Pathogenicity assessment

Pathogenicity was evaluated using *Galleria mellonella* larvae (Terraqua, Torino, Italy). Final-instar larvae (200–250 mg) were infected following the protocol by Gallorini et al. (2024). Bacterial cultures were prepared from Mueller Hinton Agar plates, then transferred to Mueller Hinton II broth and incubated at 37 °C for 16 h. Cells were centrifuged, washed in PBS, and resuspended. Optical density at 600 nm was used to standardize cell concentrations. Preliminary studies were performed with different concentrations ranging from 10^3^ to 10^8^ CFU per 10 μL. Bacterial suspensions of approx. 10^4^ CFU/10 μL were selected since they corresponded to the mortality of 50% of larvae after 1 day post inoculum (L50; data not shown) and were injected into the third left pro-leg of each larva. Serial dilutions were plated to confirm inoculum size, estimated at 5.71–5.95 log₁₀ CFU/mL. Eight strains were tested in triplicate with 10 larvae per replicate. Controls included PBS-injected larvae, non-injected larvae as well as larvae injected with a predicted hypovirulent strain (1SBR281). In total, 300 larvae were used. Infected larvae were incubated at 35 °C in darkness and monitored daily over 5 days for survival. Data were analyzed via the Kaplan–Meier estimator which was employed to determine the survival function, estimating the cumulative probability of *G. mellonella* larval survival over the five-day observation period. This non-parametric method correctly accounts for censored data (larvae that survived the full duration) by calculating the probability of survival at each observed death time based on the number of individuals at risk (Kaplan and Meier, 1958). The results are presented visually as Kaplan–Meier survival curves, and statistical differences among groups were assessed by the log-rank test using the survival package in R v4.3.2. A *p*-value < 0.05 was considered statistically significant. Moreover, pairwise comparisons between survival curves were performed using the log-rank test implemented in the survminer R package, with *p*-values adjusted for multiple testing using the Benjamini–Hochberg false discovery rate (FDR) correction.

## 3 Results

### 3.1 *Escherichia coli* taxonomic assignment and *de novo* assembly

A total of 1,170 food and environmental samples were previously collected within 6 consecutive batches in the two artisanal productions of soft cheese and salami (Pasquali et al., 2022; Pasquali et al., 2023). Based on bio-typing results, *E. coli* was detected in 33 samples: 3 from soft cheese semifinished and finished food products and 30 from salami production (raw materials, semi-finished and finished food products, environmental samples). Following whole genome sequencing, the genomes of those isolates were confirmed as belonging to *Escherichia coli* with percentages of Average Nucleotide Identity (ANI) between 98.73 and 99.94% (Table 1).

**Table 1:** *Escherichia coli* food isolates collected from cheese and salami artisanal productions.

Sample	ANI (%)	Food production	Sample origin	Batch
3SBR3	99.73	Salami	Semi-finished product	3
6 MB5	99.94	Salami	Raw material	6
5SBR5	99.94	Salami	Semi-finished product	5
4 MB1	99.88	Salami	Raw material	4
5SBR281	98.90	Salami	Finished product	5
6CP4	99.92	Cheese	Finished product	6
5SBR101	99.82	Salami	Semi-finished product	5
5STM5	98.94	Salami	Environment	5
1SBD5	98.91	Salami	Semi-finished product	1
6STM2	99.80	Salami	Environment	6
3 MB1	99.87	Salami	Raw material	3
6SWM3	99.83	Salami	Environment	6
3CP1522	99.92	Cheese	Finished product	3
2SBR183	99.82	Salami	Semi-finished product	2
3SM1	99.87	Salami	Environment	3
3SBD4	99.85	Salami	Semi-finished product	3
3CW2	99.92	Cheese	Semi-finished product	3
1SBR281	99.35	Salami	Finished product	1
4SBD3	98.73	Salami	Semi-finished product	4
4SBR281	99.79	Salami	Finished product	4
4SM3	99.71	Salami	Environment	4
4STM3	99.46	Salami	Environment	4
5 MB1	99.65	Salami	Raw material	5
6SBR282	99.83	Salami	Finished product	6
1SBR181	98.88	Salami	Semi-finished product	1
2 MB1	99.32	Salami	Raw material	2
2SBD5	99.91	Salami	Semi-finished product	2
2SBR103	99.82	Salami	Semi-finished product	2
2SBR282	99.89	Salami	Finished product	2
2SBR4	99.35	Salami	Semi-finished product	2
3SBR181	99.81	Salami	Semi-finished product	3
1SBR3	98.74	Salami	Semi-finished product	1
3SBR281	98.90	Salami	Finished product	3

Draft genomes showed good quality statistics: low number of contigs (52–172) and high N50 (91064–553,490). Genome length (4.7–5.2 Mb) and GC contents (50.4–50.8%) were in the typical range for *Escherichia coli* (Crippa et al., 2024; Criscuolo, 2018).

### 3.2 Phylogenetic analyses

**Figure 1:**
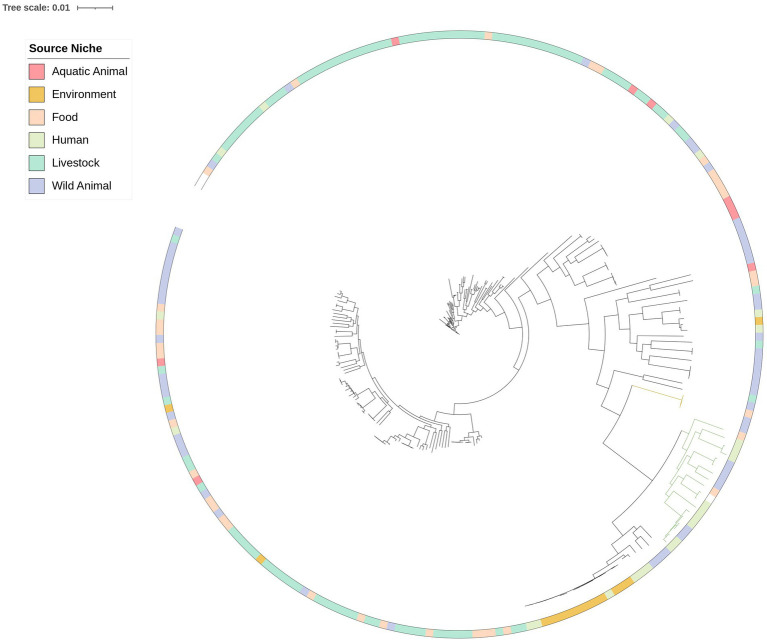
Maximum likelihood phylogenetic tree inferred from core gene alignments of 33 *Escherichia coli* (in bold) isolated from two Italian meat and dairy artisanal food productions and 202 public genomes of *E. coli* isolated in Italy from 2020 to 2023 from humans, food, animals, and the environment. The tree was rooted with reference *Escherichia coli* str. K-12 substr. MG1655 (NCBI RefSeq assembly no. GCF_000005845.2). Clusters ECO1 and ECO2 are highlighted in yellow and green branches, respectively.

In order to decipher the genetic relationship of the newly sequenced *E. coli* with isolates previously collected in Italy in diseased humans, food, animals and the environment, a maximum likelihood (ML) phylogenetic tree was inferred including 202 public genomes along with the 33 *E. coli* genomes of artisanal food origin (Figure 1). Among the 33 *E. coli* genomes high genetic distances were observed between the genomes collected from the processing environment and genomes collected from food as well as within isolates collected from food (Table 1; Figure 1; Supplementary Figure S1). In contrast, in the salami production, 3 of the five genomes of isolates collected from raw materials (pig meat mixture), namely 2 MB1, 3 MB1 and 4 MB1, showed close genetic proximity to genomes of isolates collected from food 2SBR4, 3SBD4, 2SBR282 with 26, 23 and 33 SNPs difference, respectively. This observation suggests the absence of persistent clones as well as raw material and not the processing environment as potential source of contamination of salami. Interestingly, when considering also public genomes, genome 3SBR3 (*E. coli* isolated from semifinished salami) was gathered in cluster ECO1 including also two public genomes of *E. coli* isolated from wild boar in Italy in 2021 and 2022 (assembly barcode ESC_NC7896AA_AS and ESC_NC7887AA_AS, 648 and 655 SNPs difference respectively; Figure 1; Supplementary Figure S1). This observation reinforces the suggestion of pigs and wild boars as reservoirs of *E. coli*. Moreover, genomes 6 MB5 (raw material) and 5STM5 (salami processing environment) fell within cluster ECO2 gathering 9 genomes of clinical relevance (7 isolates collected from urine, 1 from blood and 1 from wound) and 7 genomes of wild boar. Although the relevant SNP difference among genomes of this cluster (ranging from 26,260 to 37,851 SNPs difference; Figure 1; Supplementary Figure S1), the inclusion of 6 MB5 and 5STM5 in this cluster reinforces the genetic similarity of these genomes to genomes isolated from extraintestinal human sites suggesting pigs and wild boars as potential reservoirs of pathogenic *E. coli* (Figure 1).

### 3.3 Virulome

The investigation of virulence associated genes in newly sequenced *E. coli* genomes, revealed different patterns with number of virulence genes ranging from 143 to 254. Of interest, *E. coli* genomes 5STM5 and 6 MB5, which were found closely genetically related to public genomes of clinical relevance, were carriers of virulence genes belonging to ExPEC and/or aEPEC pathotypes.

**Figure 2:**
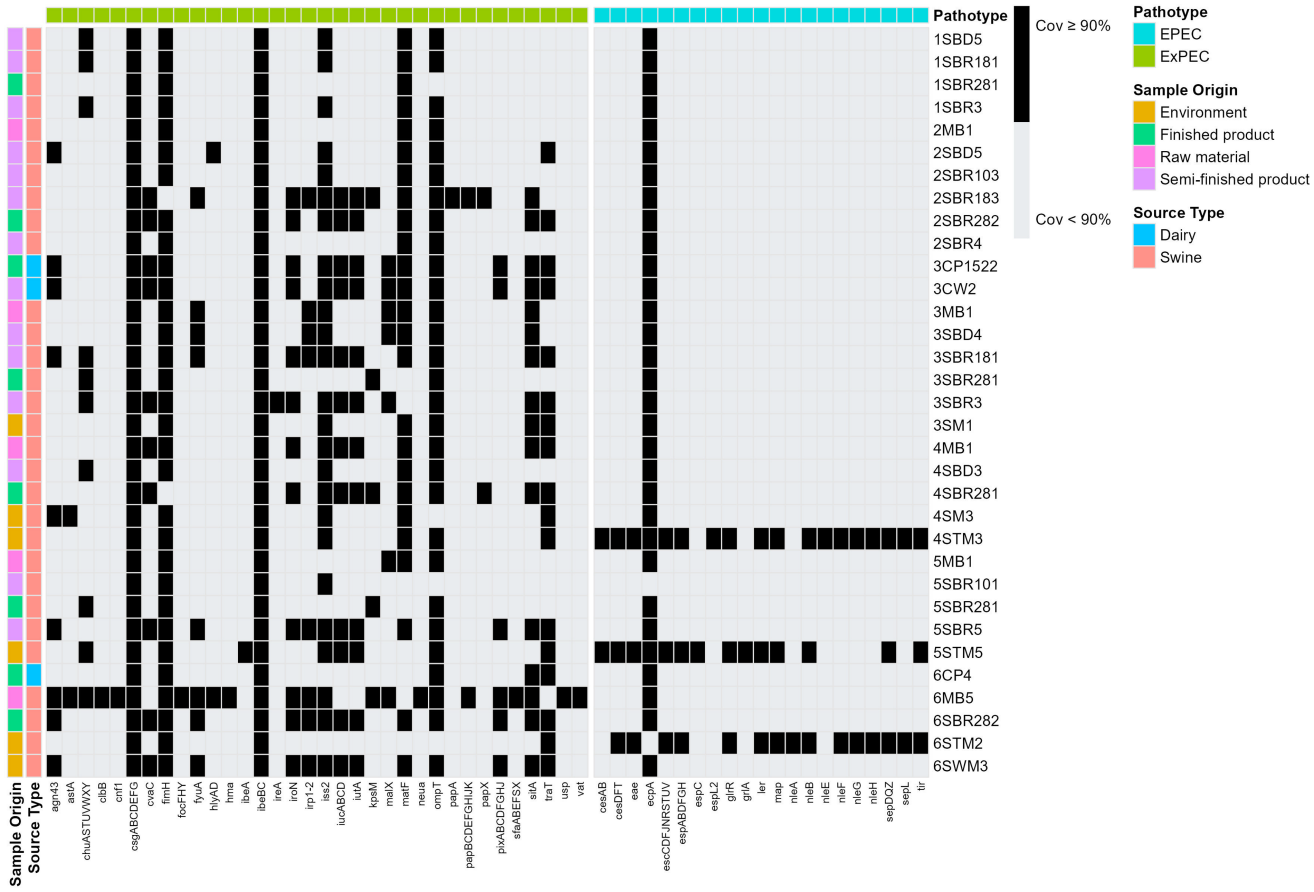
Heatmap of virulence associated genes of the 33 *E. coli* isolated from artisanal food productions. Only virulence genes already reported as associated to the EPEC (Bugarel et al., 2011; Perna et al., 2001; Garmendia et al., 2005; Karmali et al., 2003; Konczy et al., 2008) and ExPEC pathotypes (Sarowska et al., 2019; Dale and Woodford, 2015) are displayed. Colors black and white represent percentage of proportion of gene covered with reference virulence gene sequences.

In particular*, E. coli* strains 5STM5 (−: H49, ST4102), along with 4STM3 (O108: H21, ST337), and 6STM2 (O45: H2, ST301), were isolated from the processing environment of the artisanal salami (surface of the table in the stuffing room; Table 1; Figure 1). All three strains harbored the *eae* and *tir* genes within the locus of enterocyte effacement (LEE) and they were negative for the *bftp* as well as for the *stx1* and *stx2* genes thus belonging to atypical enteropathogenic *E. coli* following previous categorization (Figure 2; Levine et al., 1985). In particular they belong to phylogroups B1, F and A and to serogroups O108: H21, −: H49, and O45: H2, respectively.

Moreover, although negative for the *lifA* gene, all three genomes carried virulence genes belonging to genetic islands OI-71 (*nleH, nleA, nleF*) and OI-122 (*nleB, nleE, espL*). Interestingly, all three genomes carried additionally the *traT* gene. This gene codes for a lipoprotein located on the outer membrane of *E.coli* and involved in the adhesion to host cells.

Another interesting observation is that among genes described as virulence factor of EPEC, the *espC* gene was detected in genome 5STM5 (Figure 2; Supplementary Table S2). This genome was the only one carrying this gene among the 235 genomes analyzed, confirming the low frequency already reported previously (Afset et al., 2006; Hernandes et al., 2020; Ocampo et al., 2021).

Of interest, genome 5STM5 carries the *eae* gene along with additional genes generally listed as ExPEC virulence markers namely *chuA*, *csgA*, *ecpA*, *fimH*, *ibeA*, *iss2*, *iucA iutA, ompA, ompT* and *traT* (Sarowska et al., 2019; Dale and Woodford, 2015; Figure 2). The co-occurrence of the *eae* gene along with these genes suggest this strain as hybrid aEPEC/ExPEC. This unusual and singular genetic combination might lead to higher virulence due to the presence of virulence factors from two different pathotypes.

Along with aEPEC strains, additional attention was pointed toward 6 MB5 as well as other three *E. coli* strains: one isolated from raw materials (6 MB5 O2: H6, ST141), two from semifinished products of the ripening room (2SBR183 O5: H4, ST93; 3SBR181 D12: H4, ST57) of the salami production, and one from the cheese final product (3CP1522 O45: H8, ST 297). The genomes of these strains carried from 13 to 26 virulence genes previously reported in extraintestinal pathogenic *E. coli* such as, among others, *hlyA, cnf1, iroN, iss2, iucA, iutA, KpsM, papC* and *malX* (Sora et al., 2021; Sarowska et al., 2019; Dale and Woodford, 2015; Figure 2). The highest number of ExPEC associated virulence genes was found in *E. coli* strain 6 MB5.

### 3.4 *In vivo* pathogenicity assessment in *galleria mellonella* larvae

In order to assess the pathogenicity of presumptive aEPEC and ExPEC, *Galleria mellonella* larvae were tested as *in vivo* infection model. In particular, all *eae* and *tir* positive strains (4STM3, 5STM5 and 6STM2) were tested as presumptive aEPEC. Additionally, strains 6 MB5, 2SBR183, 3SBR181, 3CP1522 were tested by *the in vivo* approach. Although, the virulence gene pattern definition of ExPEC pathotype is still unclear, these four strains were selected for one or both of the following reasons: (1) they harbored virulence genes already described in ExPEC pathotype, (2) they showed genetic similarities with public human genomes of clinical relevance. In order to evaluate the impact on *Galleria mellonella* mortality of *E. coli* strains with different virulence patterns, and to assess the immune stimulation caused by a high bacterial load of a hypovirulent strain, the strain 1SBR281 with the lowest number of virulence genes associated to ExPEC and EPEC pathotype, was also included.

**Figure 3:**
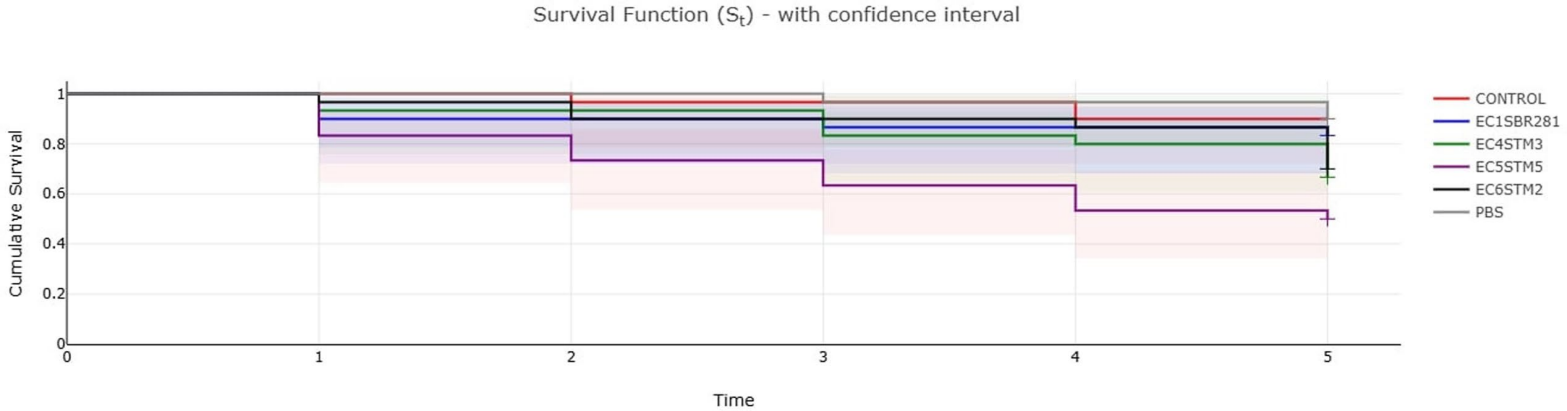
Kaplan–Meier plot showing percentage survival of *Galleria mellonella* larvae after inoculation with bacterial suspensions of *E. coli* strains representative of identified virulence patterns associated to aEPEC pathotype. Noninjected larvae (Control) and larvae injected with sterile PBS (PBS) are included. For each treatment, *n* = 30 (pooled from triplicate experiment).

After 5 days post infection, with an inoculum of 10^4^ CFU/10 μL, the highest percentage of larvae mortality among aEPEC presumptive strains, was observed in relation to challenge test with strain 5STM5. In particular, 5STM5, 4STM3 and 6STM2 were associated to survival of infected *Galleria mellonella* of 50.0%, 66.6, and 70%, respectively (Figure 3). Log-rank test analysis confirmed a statistically significant difference in survival among EPEC strains (Chi-square = 22.9, df = 5, *p* 3 × 10–4), showing from pairwise comparison 5STM5 mortality rate as significantly higher compared to 1SBR281 (*p* < 0.05; Supplementary Table S3). Actually, the predicted hypovirulent strain 1SBR281 showed indistinguishable mortality curve in comparison to not-infected larvae (CONTROL; Figure 3; Supplementary Table S3).

**Figure 4:**
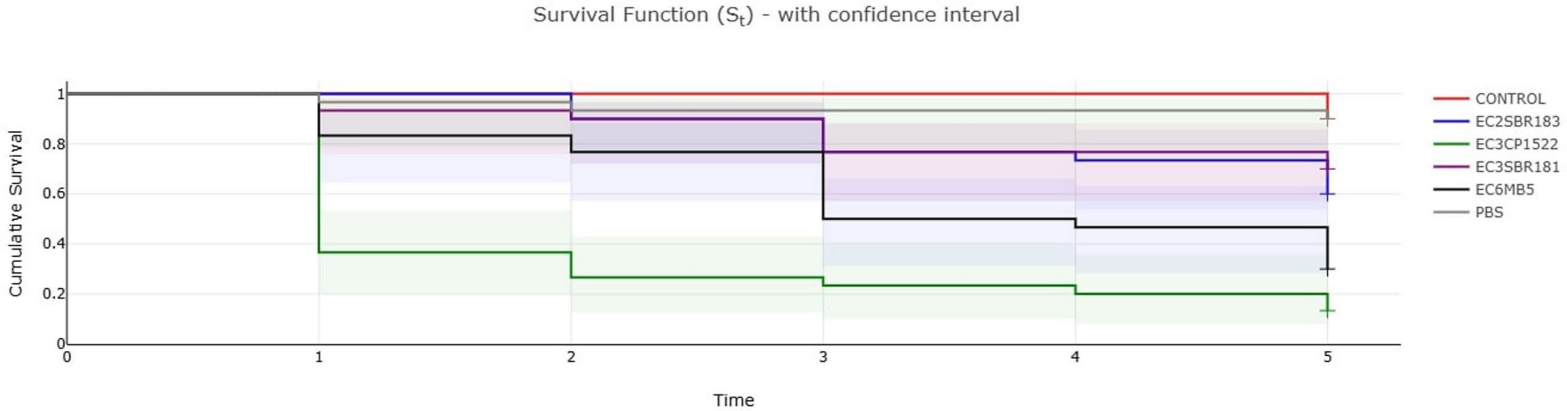
Kaplan–Meier plot showing percentage survival of *Galleria mellonella* larvae after inoculation with bacterial suspensions of *E. coli* strains representative of identified virulence patterns associated to putative ExPEC pathotype. Noninjected larvae (CONTROL) and larvae injected with sterile PBS (PBS) are included. For each treatment, *n* = 30 (pooled from triplicate experiment).

Among the presumptive ExPEC strains, the survival rates after 5 days post infection were 13,3, 30, 60 and 70% for 3CP1522, 6 MB5, 2SBR183 and 3SBR181, respectively (Figure 4), with significat difference highlighted by long-rank test (Chi-square = 92.5, df = 5, *p* < 2 × 10–16). The high mortality rate of larvae infected by 3CP1522 and 6 MB5 was statistically significant different in comparison to the mild mortality associated to 2SBR183 and 3SBR181 (*p* < 0.05; Supplementary Table S3). These results suggest the complex pathway of genotype to phenotype correspondance and it reinforces the relevance of *in vivo* models to assess aEPEC and ExPEC presumptive pathogenicity which seems not to be fully predictable by the genotype alone.

## 4 Discussion

All 33 *E. coli* isolates collected from food and food processing environments in two artisanal productions of food of animal origins were sequenced The limited number of genomes prevents any consideration on seasonal variations or environmental effects, However, these data suggest the usefulness of WGS for traceability of contamination within the food processing environment and identification of raw materials as potential sources of contamination among the salami production. Among this food production, three *E. coli* strains showed virulence patterns specific of atypical Enteropathogenic *E. coli* pathotype being *bfp*-, *stx*1-, *stx*2-, *eae*+, *tir*+. Specifically, the *eae* gene of isolates 4STM3 (O108: H21, ST337), 5STM5 (−: H49, ST4102) and 6STM2 (O45: H2, ST301), belonged to subtypes Int-*θ*, Int-*ι* and Int-*ε*, respectively. Those three subtypes are considered to have a lower occurrence in comparison to more frequently identified subtypes like Int-*β* (Ramachandran et al., 2003). The identification of putative aEPEC in artisanal salami production is a public health concern not only for food consumers but also for workers involved in the preparation of salami in the stuffing room where these pathogens were isolated. Moreover, all three genomes carried virulence genes belonging to genetic islands OI-71 (*nleH, nleA, nleF*) and OI-122 (*nleB, nleE, espL*), already associated with clinical outcomes of infection (Karmali et al., 2003; Bugarel et al., 2011; Konczy et al., 2008). Interestingly, the co-occurrence of *traT* and *eae* gene has been recently described in *E. coli* exclusively of pig origin, confirming pig as reservoir of potential aEPEC strains of specific lineage (*eae*+, *traT*+; James et al., 2025). More investigations into a higher number of strains should be performed to confirm this hypothesis.

Another interesting observation is on the *espC* gene detected in genome 5STM5, which encodes for a protein secreted independently from the type III secretion system (Mellies et al., 2001). The gene is located on a genomic island, and it is not under the regulation of the type III secretion system as other *esp* genes (Mellies et al., 2001). Protein EspC belongs to class-1 of a superfamily of virulence factors named Serine Protease Autotransporters of Enterobacteriaceae (SPATE). It acts as autotransporter and it has enterotoxin activity playing a crucial role in the cytopathic effects on intestinal epithelium during EPEC infection (Navarro-Garcia, 2023). The frequency of EspC has been described as generally low. In a previous study in Norway 8 out of 57 aEPEC strains carried the gene (Afset et al., 2006). This frequency is consistent with a more recent study which reported a frequency of 8% in aEPEC genomes from all over the World (US, Africa, Europe, South Asia; Hernandes et al., 2020). Frequencies lower than 10% were reported also in another study from Peru (Ocampo et al., 2021). Interestingly in Brazil the reported occurrence has been higher (from 28 to 49% in aEPEC and tEPEC; Abreu et al., 2013; Hernandes et al., 2020). The present study confirmed the low frequency of this gene in presumptive aEPEC as well as in other *E. coli*, with only one positive genome among the 235 Italian *E. coli* genomes analyzed. In genome 5STM5 the *espC* gene is located in a unique DNA region of the chromosome downstream of genes *glaH*, *ygaF* (renamed *lhgO*) and operon *gabDTP*. This localization has been previously reported in a *espC+* pathogenic EPEC strain E2348/69 (O127: H6; Mellies et al., 2001). Further studies should be performed to confirm the selective advantage of strains carrying the *espC* gene in the survival and pathogenicity of *E. coli* in humans.

Moreover, the co-occurrence of the *eae* gene along with extraintestinal pathogenic *E. coli* (ExPEC) virulence markers *chuA*, *csgA*, *ecpA*, *fimH*, *ibeA*, *iss2*, *iucA iutA, ompA, ompT* and *traT* (Sarowska et al., 2019; Dale and Woodford, 2015) suggest the 5STM5 strain as hybrid aEPEC/ExPEC. This unusual and singular genetic combination might lead to higher virulence due to the presence of virulence factors from two different pathotypes. Hybrid strains have been previously reported. Among others, the notable strain *E. coli* O104: H4, associated to the multi-Country outbreak of 2011, was a hybrid pathogen that carried virulence genes found in both typical enteroaggregative *E. coli* (EAEC) strains (*aggA, aggR, set1, pic, and aap*) and Shiga-toxin producing *E. coli* (STEC; *stx 2*; Croxen et al., 2013). More specifically, a hybrid aEPEC/ExPEC isolate was collected in Brazil from feces of a patient with acute diarrhea and firstly assigned as EPEC. After sequencing, the genome of the isolate revealed the presence of LEE genes along with 16 virulence genes more frequently identified in ExPEC strains (Munhoz et al., 2021). Another hybrid aEPEC/UPEC was collected from patient with urinary tract infection in Italy and firstly assigned as UPEC. After sequencing, authors noticed the presence of the LEE, which genes *espB* and *eae* were confirmed as expressed (Valiatti et al., 2020).

Concerning the *in vivo* pathogenicity assessment in *G. mellonella* larvae, results of survival rates reinforced the genomic predictions of higher virulence of aEPEC strain 5STM5 and pointed out to an absent or mild pathogenic potential of aEPEC strains 4STM3 and 6STM2. The mild pathogenicity potential of strain 4STM3, is reinforced by its phylogenetic proximity to the human strain with assembly barcode ESC_PC5093AA_AS isolated previously from a healthy donor (Figure 1; Di Pierro et al., 2024). Mild to non-pathogenic aEPEC have been already described worldwide (Hernandes et al., 2009; Nguyen et al., 2006). On the other hand, the mild pathogenicity encountered for 6STM2 is surprising since this strain belongs to ST301 clonal complex and to serogroup 045: H2 already described as gathering shiga toxin producing *Escherichia coli* (Zhang et al., 2020).

Along with aEPEC putative strains, four strains showed interesting virulence patterns being positive for several genes already described in ExPEC pathotypes. Although virulence genes of ExPEC have been described, there is not at present an agreed identification of key virulence markers essential for the observation of this pathotype, making difficult the prediction of the pathogenic potential of these strains solely based on their virulome.

Among the presumptive ExPEC strains, the two with the highest mortality rate in *G. mellonella* larvae (3CP1522 and 6 MB5) carried the *malX* gene which was absent in the genomes of the other strains tested. This gene is a virulence marker of the pathogenicity Island of strain CFT073 and has been described as an epidemiological predictor of ExPEC specifically of urinary and pulmonary source (Johnson and Russo, 2018; Johnson et al., 2002). Additionally, it was found in an epidemic clone of multiresistant ESBL ExPEC in Denmark (Olesen et al., 2013). In the present study the *malX* gene showed a significantly higher occurrence in humans vs. other (food, environmental, animal) sources (65% vs. 17%, *p*-value = 4.958e-07), substantiating its role in ExPEC pathotype. The 6 MB5 genome carried additionally the *cnf* gene coding for the cytotoxic necrotizing factor (CNF), which has been traditionally considered as a ExPEC virulence gene marker thanks also to confirmatory evidence in animal model infection studies (Khan et al., 2002; Rippere-Lampe et al., 2001). As for *malX*, in the present study also the occurrence of *cnf1* gene was higher in isolates of clinical origin *vs* other origins (30% *vs* 3%) although this difference was not statistically significant (p-value = 0.2466). Surprisingly, although representing the genome with the highest number of ExPEC virulence strains, 6 MB5 strain was not the strain with the highest pathogenicity in *G. mellonella* reinforcing the complexity of ExPEC phenotype prediction based on the virulome. Whole genome sequencing is effective in detecting virulence genes but is not informative on their expression. A phenotypic virulence might be affected by gene regulation, non-functional genes or complex interactions among genes and proteins. Genotypic to phenotypic discordances have been fully described for antimicrobial resistance (Yee et al., 2021). These discrepancies further reinforce the relevance of an *in vivo* infection model to phenotypically explore the pathogenicity potential of strains with specific virulence patterns. This study has limitations. Along with the limited number of strains tested, results on an *in-vivo* infection model should be considered with caution when translability to humans is considered. Moreover, concerning the highlighted *espC*, *malX* and *cnf1* genes, a specific quantitative risk assessment is required to evaluate the real impact on consumer health risk. Further studies are needed to confirm the relevance of these genes on aEPEC and ExPEC pathotypes.

## 5 Conclusion

In conclusion, the combined genomic and *in vivo* approach used in the present study was useful to evaluate the potential pathogenicity of *E. coli* strains isolated from artisanal salami and soft cheese helping in pinpointing to specific aEPEC, ExPEC and hybrid aEPEC/ExPEC strains which showed higher mortality in *G. mellonella* larvae and were characterized by additional virulence genes such as *eae* and *espC* in putative aEPEC, and *malX* in putative ExPEC. The potential concern for public health associated to these strains, and in particular the direct role of each of these genes alone and in combination with others, is worthy of future investigations which could help to assess the risk for consumers after the consumption of contaminated artisanal food.

## Data Availability

The paired-end reads for this study can be found in the European Nucleotide Archive (ENA) at EMBL-EBI under accession number PRJEB71359 (https://www.ebi.ac.uk/ena/browser/view/PRJEB71359).

## Supplementary material

SUPPLEMENTARY TABLE S1 *Escherichia coli* public genomes of human, food, animal and environmental origin included in this study.

SUPPLEMENTARY TABLE S2 Matrix of virulence genes of the 235 *Escherichia coli* genomes included in this study.

SUPPLEMENTARY TABLE S3 Pairwise log-rank test results comparing larval survival among EPEC and ExPEC isolates and control groups. Adjusted *p*-values were calculated using the Benjamini–Hochberg method (FDR). Significance codes: ****p* < 0.001, ***p* < 0.01, **p* < 0.05, ns, not significant.

SUPPLEMENTARY FIGURE S1 Heatmap of pairwise SNP distance matrix of the 33 *Escherichia coli* food genomes and public genomes belonging to phylogenetic clusters ECO1 and ECO2.

## References

[ref1] Abreu A. G. Bueris V. Porangaba T. M. Sircili M. P. Navarro-Garcia F. Elias W. P. (2013). Autotransporter protein-encoding genes of diarrheagenic *Escherichia coli* are found in both typical and atypical enteropathogenic *E. coli* strains. Appl. Eviron. Microbiol. 79, 411–414. doi: 10.1128/AEM.02635-12, 23104414 PMC3536084

[ref2] Abri R. Javadi A. Asghari R. Razavilar V. Salehi T. Z. Safaeeyan F. . (2019). Surveillance for enterotoxigenic and enteropathogenic *Escherichia coli* isolates from animal source foods in Northwest Iran. Indian J. Med. Res. 150, 87–91. doi: 10.4103/ijmr.IJMR_2019_17, 31571634 PMC6798612

[ref3] Afset J. E. Bruant G. Brousseau R. Harel J. Anderssen E. Bevanger L. . (2006). Identification of virulence genes linked with diarrhea due to atypical enteropathogenic *Escherichia coli* by DNA microarray analysis and PCR. J. Clin. Microbiol. 44, 3703–3711. doi: 10.1128/JCM.00429-06, 17021100 PMC1594803

[ref4] Almli V. L. Verbeke W. Vanhonacker F. Næs T. Hersleth M. (2011). General image and attribute perceptions of traditional food in six European countries. Food Qual. Prefer. 22, 129–138. doi: 10.1016/j.foodqual.2010.08.008

[ref5] Alonso M. Z. Padola N. L. Parma A. E. Lucchesi P. M. (2011). Enteropathogenic *Escherichia coli* contamination at different stages of the chicken slaughtering process. Poult. Sci. 90, 2638–2641. doi: 10.3382/ps.2011-01621, 22010252

[ref6] Anjum M. F. Schmitt H. Börjesson S. Berendonk T. U. WAWES network (2021). The potential of using *E. coli* as an indicator for the surveillance of antimicrobial resistance (AMR) in the environment. Curr. Opin. Microbiol. 64, 152–158. doi: 10.1016/j.mib.2021.09.011, 34739920

[ref7] Antão E. M. Glodde S. Li G. Sharifi R. Homeier T. Laturnus C. . (2008). The chicken as a natural model for extraintestinal infections caused by avian pathogenic *Escherichia coli* (APEC). Microb. Pathog. 45, 361–369. doi: 10.1016/j.micpath.2008.08.005, 18848980

[ref8] Assolatte. (2025). Rapporto ASSOLATTE 2025. Available online at: https://www.assolatte.it/it/home/news_detail/attualita/1751433709593 [Accessed on the 24th October 2025]

[ref9] Bergeron C. R. Prussing C. Boerlin P. Daignault D. Dutil L. Reid-Smith R. J. . (2012). Chicken as reservoir for extraintestinal pathogenic *Escherichia coli* in humans. Canada. Emerg. Infect. Dis. 18, 415–421. doi: 10.3201/eid1803.111099, 22377351 PMC3309577

[ref10] Bortolaia V. Kaas R. S. Ruppe E. Roberts M. C. Schwarz S. Cattoir V. . (2020). ResFinder 4.0 for predictions of phenotypes from genotypes. J. Antimicrob. Chemother. 75, 3491–3500. doi: 10.1093/jac/dkaa345, 32780112 PMC7662176

[ref11] Bugarel M. Martin A. Fach P. Beutin L. (2011). Virulence gene profiling of enterohemorrhagic (EHEC) and enteropathogenic (EPEC) *Escherichia coli* strains: a basis for molecular risk assessment of typical and atypical EPEC strains. BMC Microbiol. 11:142. doi: 10.1186/1471-2180-11-142, 21689465 PMC3133550

[ref12] Chen L. Zheng D. Liu B. Yang J. Jin Q. (2016). VFDB 2016: hierarchical and refined dataset for big data analysis—10 years on. Nucleic Acids Res. 44, D694–D697. doi: 10.1093/nar/gkv1239, 26578559 PMC4702877

[ref13] Cirne C. T. Tunick M. H. Trout R. E. (2019). The chemical and attitudinal differences between commercial and artisanal products. NPJ Sci. Food 3:19. doi: 10.1038/s41538-019-0053-9, 31508494 PMC6731266

[ref14] Crippa C. De Cesare A. Lucchi A. Parisi A. Manfreda G. Pasquali F. (2024). Occurrence and genomic characterization of antimicrobial-resistant and potential pathogenic *Escherichia coli* from Italian artisanal food productions of animal origin. Ital. J. Food Saf. 13:12205. doi: 10.4081/ijfs.2024.12205, 38846048 PMC11154171

[ref15] Criscuolo A. (2018). Contig_info - estimating standard descriptive statistics from contig sequences (version 2.0.1). Available online at: https://gitlab.pasteur.fr/GIPhy/contig_info [Accessed August 5, 2025)].

[ref16] Croxen M. A. Law R. J. Scholz R. Keeney K. M. Wlodarska M. Finlay B. B. (2013). Recent advances in understanding enteric pathogenic *Escherichia coli*. Clin. Microbiol. Rev. 26, 822–880. doi: 10.1128/CMR.00022-13, 24092857 PMC3811233

[ref17] Dale A. P. Woodford N. (2015). Extra-intestinal pathogenic *Escherichia coli* (ExPEC): disease, carriage and clones. J. Inf. Secur. 71, 615–626. doi: 10.1016/j.jinf.2015.09.009, 26409905

[ref18] Di Pierro F. Zerbinati N. Guasti L. Cazzaniga M. Bertuccioli A. Palazzi C. M. . (2024). Draft genome sequence of non-pathogenic *Escherichia coli* 5C LMG S-33222, isolated from healthy donor feces. Microbiol. Resour. Announc. 13:e0058024. doi: 10.1128/mra.00580-24, 39283993 PMC11465944

[ref20] Dyer N. P. Päuker B. Baxter L. Gupta A. Bunk B. Overmann J. . (2025). EnteroBase in 2025: exploring the genomic epidemiology of bacterial pathogens. Nucleic Acids Res. 53, D757–D762. doi: 10.1093/nar/gkae902, 39441072 PMC11701629

[ref21] ECDC, EFSA Panel on Biological Hazards (BIOHAZ), and EMA Committee for Medicinal Products for Veterinary Use (CVMP) (2017). ECDC, EFSA and EMA joint scientific opinion on a list of outcome indicators as regards surveillance of antimicrobial resistance and antimicrobial consumption in humans and food-producing animals. EFSA J. 15:e05017. doi: 10.2903/j.efsa.2017.5017, 32625307 PMC7009961

[ref23] Eurispes (2017). 29° Rapporto Italia. Bologna, Italy: Minerva Edizioni.

[ref24] Gallorini M. Marinacci B. Pellegrini B. Cataldi A. Dindo M. L. Carradori S. . (2024). Immunophenotyping of hemocytes from infected galleria mellonella larvae as an innovative tool for immune profiling, infection studies and drug screening. Sci. Rep. 14:759. doi: 10.1038/s41598-024-51316-z, 38191588 PMC10774281

[ref25] Garmendia J. Frankel G. Crepin V. F. (2005). Enteropathogenic and enterohemorrhagic *Escherichia coli* infections: translocation, translocation, translocation. Infect. Immun. 73, 2573–2585. doi: 10.1128/IAI.73.5.2573-2585.2005, 15845459 PMC1087358

[ref26] Gomes T. A. Elias W. P. Scaletsky I. C. Guth B. E. Rodrigues J. F. Piazza R. M. . (2016). Diarrheagenic *Escherichia coli*. Braz. J. Microbiol. 47, 3–30. doi: 10.1016/j.bjm.2016.10.015, 27866935 PMC5156508

[ref27] Green S. I. Ajami N. J. Ma L. Poole N. M. Price R. E. Petrosino J. F. . (2015). Murine model of chemotherapy-induced extraintestinal pathogenic *Escherichia coli* translocation. Infect. Immun. 83, 3243–3256. doi: 10.1128/IAI.00684-15, 26034214 PMC4496622

[ref28] Guindon S. Dufayard J. F. Lefort V. Anisimova M. Hordijk W. Gascuel O. (2010). New algorithms and methods to estimate maximum-likelihood phylogenies: assessing the performance of PhyML 3.0. Syst. Biol. 59, 307–321. doi: 10.1093/sysbio/syq010, 20525638

[ref29] Hernandes R. T. Elias W. P. Vieira M. A. Gomes T. A. (2009). An overview of atypical enteropathogenic *Escherichia coli*. FEMS Microbiol. Lett. 297, 137–149. doi: 10.1111/j.1574-6968.2009.01664.x, 19527295

[ref30] Hernandes R. T. Hazen T. H. Dos Santos L. F. Richter T. K. S. Michalski J. M. Rasko D. A. (2020). Comparative genomic analysis provides insight into the phylogeny and virulence of atypical enteropathogenic *Escherichia coli* strains from Brazil. PLoS Negl. Trop. Dis. 14:e0008373. doi: 10.1371/journal.pntd.0008373, 32479541 PMC7289442

[ref32] Jakobsen L. Garneau P. Bruant G. Harel J. Olsen S. S. Porsbo L. J. . (2012). Is *Escherichia coli* urinary tract infection a zoonosis? Proof of direct link with production animals and meat. Eur. J. Clin. Microbiol. Infect. Dis. 31, 1121–1129. doi: 10.1007/s10096-011-1417-5, 22033854

[ref33] James E. M. Kimera Z. I. Mgaya F. X. Niccodem E. M. Efraim J. E. Matee M. I. . (2025). Occurrence of virulence genes in multidrug-resistant *Escherichia coli* isolates from humans, animals, and the environment: one health perspective. PLoS One 20:e0317874. doi: 10.1371/journal.pone.0317874, 39854442 PMC11760637

[ref34] Jaramillo-Bedoya E. Trujillo-Alzate Y. A. Ocampo-Ibáñez I. D. (2021). Surveillance of fresh artisanal cheeses revealed high levels of *Listeria monocytogenes* contamination in the Department of Quindío, Colombia. Pathogens 10:1341. doi: 10.3390/pathogens10101341, 34684290 PMC8537478

[ref35] Johnson J. R. Kuskowski M. A. O'Bryan T. T. Maslow J. N. (2002). Epidemiological correlates of virulence genotype and phylogenetic background among *Escherichia coli* blood isolates from adults with diverse-source bacteremia. J. Infect. Dis. 185, 1439–1447. doi: 10.1086/340506, 11992279

[ref36] Johnson J. R. Russo T. A. (2018). Molecular epidemiology of extraintestinal pathogenic *Escherichia coli*. EcoSal Plus 8:2017. doi: 10.1128/ecosalplus.ESP-0004-2017, 29667573 PMC11575673

[ref37] Jolley K. A. Maiden M. C. (2010). BIGSdb: scalable analysis of bacterial genome variation at the population level. BMC Bioinformatics 11:595. doi: 10.1186/1471-2105-11-595, 21143983 PMC3004885

[ref38] Kaper J. B. Nataro J. P. Mobley H. L. (2004). Pathogenic *Escherichia coli*. Nat. Rev. Microbiol. 2, 123–140. doi: 10.1038/nrmicro818 15040260

[ref39] Kaplan E. L. Meier P. (1958). Nonparametric estimation from incomplete observations. J. Am. Stat. Assoc. 53, 457–481.

[ref40] Karmali M. A. Mascarenhas M. Shen S. Ziebell K. Johnson S. Reid-Smith R. . (2003). Association of genomic O island 122 of *Escherichia coli* EDL 933 with verocytotoxin-producing *Escherichia coli* seropathotypes that are linked to epidemic and/or serious disease. J. Clin. Microbiol. 41, 4930–4940. doi: 10.1128/JCM.41.11.4930-4940.2003, 14605120 PMC262514

[ref41] Khan N. A. Wang Y. Kim K. J. Chung J. W. Wass C. A. Kim K. S. (2002). Cytotoxic necrotizing factor-1 contributes to *Escherichia coli* K1 invasion of the central nervous system. J. Biol. Chem. 277, 15607–15612. doi: 10.1074/jbc.M112224200, 11877402

[ref42] Kinnula S. Hemminki K. Kotilainen H. Ruotsalainen E. Tarkka E. Salmenlinna S. . (2018). Outbreak of multiple strains of non-O157 Shiga toxin-producing and enteropathogenic *Escherichia coli* associated with rocket salad, Finland, autumn 2016. Euro Surveill. 23:1700666. doi: 10.2807/1560-7917.ES.2018.23.35.1700666, 30180926 PMC6124187

[ref43] Konczy P. Ziebell K. Mascarenhas M. Choi A. Michaud C. Kropinski A. M. . (2008). Genomic O island 122, locus for enterocyte effacement, and the evolution of virulent verocytotoxin-producing *Escherichia coli*. J. Bacteriol. 190, 5832–5840. doi: 10.1128/JB.00480-08, 18586943 PMC2519529

[ref44] Lee J. B. Kim S. K. Yoon J. W. (2022). Pathophysiology of enteropathogenic *Escherichia coli* during a host infection. J. Vet. Sci. 23:e28. doi: 10.4142/jvs.21160, 35187883 PMC8977535

[ref45] Letunic I. Bork P. (2024). Interactive tree of life (iTOL) v6: recent updates to the phylogenetic tree display and annotation tool. Nucleic Acids Res. 52, W78–W82. doi: 10.1093/nar/gkae268, 38613393 PMC11223838

[ref46] Leuko S. Raivio T. L. (2012). Mutations that impact the enteropathogenic *Escherichia coli* Cpx envelope stress response attenuate virulence in galleria mellonella. Infect. Immun. 80, 3077–3085. doi: 10.1128/IAI.00081-12, 22710873 PMC3418753

[ref47] Levine M. M. Nataro J. P. Karch H. Baldini M. M. Kaper J. B. Black R. E. . (1985). The diarrheal response of humans to some classic serotypes of enteropathogenic *Escherichia coli* is dependent on a plasmid encoding an enteroadhesiveness factor. J. Infect. Dis. 152, 550–559. 2863318 10.1093/infdis/152.3.550

[ref48] Lim M. A. Kim J. Y. Acharya D. Bajgain B. B. Park J. H. Yoo S. J. . (2020). A Diarrhoeagenic Enteropathogenic *Escherichia coli* (EPEC) infection outbreak that occurred among elementary school children in Gyeongsangbuk-Do Province of South Korea was associated with consumption of water-contaminated food items. Int. J. Environ. Res. Public Health 17:3149. doi: 10.3390/ijerph17093149, 32366011 PMC7246572

[ref49] Manges A. R. Johnson J. R. (2012). Food-borne origins of *Escherichia coli* causing extraintestinal infections. Clin. Infect. Dis. 55, 712–719. doi: 10.1093/cid/cis502, 22615330

[ref50] Meena P. R. Priyanka P. Singh A. P. (2023). Extraintestinal pathogenic *Escherichia coli* (ExPEC) reservoirs, and antibiotics resistance trends: a one-health surveillance for risk analysis from "farm-to-fork". Lett. Appl. Microbiol. 76:ovac016. doi: 10.1093/lambio/ovac016, 36688760

[ref51] Mellies J. L. Navarro-Garcia F. Okeke I. Frederickson J. Nataro J. P. Kaper J. B. (2001). espC pathogenicity island of enteropathogenic *Escherichia coli* encodes an enterotoxin. Infect. Immun. 69, 315–324. doi: 10.1128/IAI.69.1.315-324.2001, 11119520 PMC97886

[ref52] Ménard G. Rouillon A. Cattoir V. Donnio P. Y. (2021). Galleria mellonella as a suitable model of bacterial infection: past, present and future. Front. Cell. Infect. Microbiol. 11:782733. doi: 10.3389/fcimb.2021.782733, 35004350 PMC8727906

[ref53] Munhoz D. D. Santos F. F. Mitsunari T. Schüroff P. A. Elias W. P. Carvalho E. . (2021). Hybrid atypical Enteropathogenic and Extraintestinal *Escherichia coli* (aEPEC/ExPEC) BA1250 strain: a draft genome. Pathogens (Basel, Switzerland) 10:475. doi: 10.3390/pathogens10040475, 33919948 PMC8070890

[ref54] Navarro-Garcia F. (2023). Serine proteases autotransporter of Enterobacteriaceae: structures, subdomains, motifs, functions, and targets. Mol. Microbiol. 120, 178–193. doi: 10.1111/mmi.15116, 37392318

[ref56] Nguyen R. N. Taylor L. S. Tauschek M. Robins-Browne R. M. (2006). Atypical enteropathogenic *Escherichia coli* infection and prolonged diarrhea in children. Emerg. Infect. Dis. 12, 597–603. doi: 10.3201/eid1204.051112, 16704807 PMC3294699

[ref57] Ocampo K. Riveros M. Pinedo-Bardales M. Ruiz J. Ochoa T. J. (2021). Frequency of serine protease autotransporters of enterobacteriaceae (SPATE) encoding genes in diffusely adherent *escherichia coli* (DAEC) isolates from children with and without diarrhea. Frecuencia de genes que codifican proteínas autotransportadoras serin-proteasa de Enterobacteriaceae (SPATE) en cepas de Escherichia coli difusamente adherente (DAEC) provenientes de niños con y sin diarrea. Rev. Peru Med. Exp. Salud Publica 38, 124–129. doi: 10.17843/rpmesp.2021.381.5750 34190904

[ref58] Ochoa T. J. Contreras C. A. (2011). Enteropathogenic *Escherichia coli* infection in children. Curr. Opin. Infect. Dis. 24, 478–483. doi: 10.1097/QCO.0b013e32834a8b8b, 21857511 PMC3277943

[ref60] Olesen B. Hansen D. S. Nilsson F. Frimodt-Møller J. Leihof R. F. Struve C. . (2013). Prevalence and characteristics of the epidemic multiresistant *Escherichia coli* ST131 clonal group among extended-spectrum beta-lactamase-producing *E. coli* isolates in Copenhagen, Denmark. J. Clin. Microbiol. 51, 1779–1785. doi: 10.1128/JCM.00346-13, 23554186 PMC3716056

[ref61] Onlen Guneri C. Koksal F. Kizilyildirim S. Bedir B. Nagiyev T. (2022). The distribution of cytotoxic necrotizing factors (CNF-1, CNF-2, CNF-3) and cytolethal distending toxins (CDT-1, CDT-2, CDT-3, CDT-4) in *Escherichia coli* isolates isolated from extraintestinal infections and the determination of their phylogenetic relationship by PFGE. Int. J. Clin. Pract. 2022:7200635. doi: 10.1155/2022/7200635, 36474550 PMC9683945

[ref62] Pasquali F. Crippa C. Lucchi A. Francati S. Dindo M. L. Manfreda G. (2025). *Citrobacter braakii* isolated from salami and soft cheese: an emerging food safety hazard? Foods 14:1887. doi: 10.3390/foods14111887, 40509415 PMC12154454

[ref63] Pasquali F. Valero A. Possas A. Lucchi A. Crippa C. Gambi L. . (2022). Occurrence of foodborne pathogens in Italian soft artisanal cheeses displaying different intra- and inter-batch variability of physicochemical and microbiological parameters. Front. Microbiol. 13:959648. doi: 10.3389/fmicb.2022.959648, 36090085 PMC9453248

[ref64] Pasquali F. Valero A. Possas A. Lucchi A. Crippa C. Gambi L. . (2023). Variability in physicochemical parameters and its impact on microbiological quality and occurrence of foodborne pathogens in artisanal Italian organic salami. Foods 12:4086. doi: 10.3390/foods12224086, 38002143 PMC10670534

[ref65] Peirano G. Mulvey G. L. Armstrong G. D. Pitout J. D. D. (2013). Virulence potential and adherence properties of *Escherichia coli* that produce CTX-M and NDM β-lactamases. J. Med. Microbiol. 62, 525–530. doi: 10.1099/jmm.0.048983-0, 23319311

[ref66] Perna N. T. Plunkett G. Burland V. Mau B. Glasner J. D. Rose D. J. . (2001). Genome sequence of enterohaemorrhagic *Escherichia coli* O157:H7. Nature 409, 529–533. doi: 10.1038/35054089 11206551

[ref67] Platenkamp A. Mellies J. L. (2018). Environment controls LEE regulation in Enteropathogenic *Escherichia coli*. Front. Microbiol. 9:1694. doi: 10.3389/fmicb.2018.01694, 30140259 PMC6094958

[ref69] Ramachandran V. Brett K. Hornitzky M. A. Dowton M. Bettelheim K. A. Walker M. J. . (2003). Distribution of intimin subtypes among *Escherichia coli* isolates from ruminant and human sources. J. Clin. Microbiol. 41, 5022–5032. doi: 10.1128/JCM.41.11.5022-5032.2003, 14605134 PMC262460

[ref70] Ramchandani M. Manges A. R. DebRoy C. Smith S. P. Johnson J. R. Riley L. W. (2005). Possible animal origin of human-associated, multidrug-resistant, uropathogenic *Escherichia coli*. Clin. Infect. Dis. 40, 251–257. doi: 10.1086/426819, 15655743

[ref71] Rehman M. U. Zhang H. Wang Y. Mehmood K. Huang S. Iqbal M. K. . (2017). Experimental mouse lethality of *Escherichia coli* strains isolated from free ranging Tibetan yaks. Microb. Pathog. 109, 15–19. doi: 10.1016/j.micpath.2017.05.020, 28506886

[ref72] Rippere-Lampe K. E. Lang M. Ceri H. Olson M. Lockman H. A. O’Brien A. D. (2001). Cytotoxic necrotizing factor type 1-positive *Escherichia coli* causes increased inflammation and tissue damage to the prostate in a rat prostatitis model. Infect. Immun. 69, 6515–6519. doi: 10.1128/IAI.69.10.6515-6519.2001, 11553597 PMC98788

[ref74] Roccato A. Uyttendaele M. Barrucci F. Cibin V. Favretti M. Cereser A. . (2017). Artisanal Italian salami and soppresse: identification of control strategies to manage microbiological hazards. Food Microbiol. 61, 5–13. doi: 10.1016/j.fm.2016.07.010, 27697168

[ref75] Russo T. A. Johnson J. R. (2003). Medical and economic impact of extraintestinal infections due to *Escherichia coli*: focus on an increasingly important endemic problem. Microbes Infect. 5, 449–456. doi: 10.1016/s1286-4579(03)00049-2, 12738001

[ref76] Sarowska J. Futoma-Koloch B. Jama-Kmiecik A. Frej-Madrzak M. Ksiazczyk M. Bugla-Ploskonska G. . (2019). Virulence factors, prevalence and potential transmission of extraintestinal pathogenic *Escherichia coli* isolated from different sources: recent reports. Gut Pathog. 11:10. doi: 10.1186/s13099-019-0290-0, 30828388 PMC6383261

[ref78] Seemann T. (2015). Rapid haploid variant calling and core genome alignment (version 4.6.0). Available online at: https://github.com/tseemann/snippy [Accessed August 5, 2025].

[ref79] Seemann T. (2020). Abricate - mass screening of contigs for antimicrobial resistance or virulence genes (version 1.0.1). Available online at: https://github.com/tseemann/abricate [Accessed August 5, 2025].

[ref80] Seemann T. (2022). MLST- scan contig files against traditional PubMLST typing schemes (version 2.23.0). Available online at: https://github.com/tseemann/mlst [Accessed August 5, 2025].

[ref81] Serrano I. Verdial C. Tavares L. Oliveira M. (2023). The Virtuous Galleria mellonella Model for Scientific Experimentation. Antibiotics (Basel, Switzerland) 12:505. doi: 10.3390/antibiotics12030505, 36978373 PMC10044286

[ref82] Slinger R. Lau K. Slinger M. Moldovan I. Chan F. (2017). Higher atypical enteropathogenic *Escherichia coli* (a-EPEC) bacterial loads in children with diarrhea are associated with PCR detection of the EHEC factor for adherence 1/lymphocyte inhibitory factor a (efa1/lifa) gene. Ann. Clin. Microbiol. Antimicrob. 16:16. doi: 10.1186/s12941-017-0188-y, 28330478 PMC5363046

[ref84] Sora V. M. Meroni G. Martino P. A. Soggiu A. Bonizzi L. Zecconi A. (2021). Extraintestinal pathogenic *Escherichia coli*: virulence factors and antibiotic resistance. Pathogens (Basel, Switzerland) 10:1355. doi: 10.3390/pathogens10111355, 34832511 PMC8618662

[ref85] Taha Z. M. Yassin N. A. (2019). Prevalence of diarrheagenic *Escherichia coli* in animal products in Duhok province, Iraq. Iran. J. Vet. Res. 20, 255–262. doi: 10.22099/ijvr.2019.5502 32042289 PMC6983314

[ref86] Taouk M. L. Featherstone L. A. Taiaroa G. Seemann T. Ingle D. J. Stinear T. P. . (2025). Exploring SNP filtering strategies: the influence of strict vs soft core. Microb. Genom. 11:e001346. doi: 10.1099/mgen.0.001346, 39812553 PMC11734701

[ref87] Trobos M. Christensen H. Sunde M. Nordentoft S. Agersø Y. Simonsen G. S. . (2009). Characterization of sulphonamide-resistant *Escherichia coli* using comparison of sul2 gene sequences and multilocus sequence typing. Microbiology 155, 831–836. doi: 10.1099/mic.0.024190-0, 19246754

[ref88] Valiatti T. B. Santos F. F. Santos A. C. M. Nascimento J. A. S. Silva R. M. Carvalho E. . (2020). Genetic and virulence characteristics of a hybrid atypical Enteropathogenic and Uropathogenic *Escherichia coli* (aEPEC/UPEC) strain. Front. Cell. Infect. Microbiol. 10:492. doi: 10.3389/fcimb.2020.00492, 33134184 PMC7550682

[ref89] Velikova N. Kavanagh K. Wells J. M. (2016). Evaluation of galleria mellonella larvae for studying the virulence of *Streptococcus suis*. BMC Microbiol. 16:291. doi: 10.1186/s12866-016-0905-2, 27978817 PMC5160000

[ref90] Vincent C. Boerlin P. Daignault D. Dozois C. M. Dutil L. Galanakis C. . (2010). Food reservoir for *Escherichia coli* causing urinary tract infections. Emerg. Infect. Dis. 16, 88–95. doi: 10.3201/eid1601.091118, 20031048 PMC2874376

[ref91] Wick R. R. Judd L. M. Gorrie C. L. Holt K. E. (2017). Unicycler: resolving bacterial genome assemblies from short and long sequencing reads. PLoS Comput. Biol. 13:e1005595. doi: 10.1371/journal.pcbi.1005595, 28594827 PMC5481147

[ref92] Williamson D. A. Mills G. Johnson J. R. Porter S. Wiles S. (2014). In vivo correlates of molecularly inferred virulence among extraintestinal pathogenic *Escherichia coli* (ExPEC) in the wax moth galleria mellonella model system. Virulence 5, 388–393. doi: 10.4161/viru.27912, 24518442 PMC3979865

[ref93] Yee R. Dien Bard J. Simner P. J. (2021). The genotype-to-phenotype dilemma: how should laboratories approach discordant susceptibility results? J. Clin. Microbiol. 59, e00138–e00120. doi: 10.1128/JCM.00138-20, 33441396 PMC8316082

[ref94] Zhang Y. Liao Y. T. Sun X. Wu V. C. H. (2020). Is Shiga toxin-producing *Escherichia coli* O45 no longer a food safety threat? The danger is still out there. Microorganisms 8:782. doi: 10.3390/microorganisms8050782, 32455956 PMC7285328

[ref95] Zhou Z. Alikhan N.-F. Mohamed K. Fan Y. the Agama Study Group Achtman M. (2020). The EnteroBase user’s guide, with case studies on *Salmonella* transmissions, *Yersinia pestis* phylogeny, and *Escherichia* core genomic diversity. Genome Res. 30, 138–152. doi: 10.1101/gr.251678.119, 31809257 PMC6961584

