# Supplementary figures and images for: Artisanal food of animal origin as reservoir of putative pathogenic Escherichia coli: a combined genomic and in vivo approach
Source: Front Microbiol. 2026 Jan 21;16:1718380. doi: 10.3389/fmicb.2025.1718380 (PMC12868146; doi:10.3389/fmicb.2025.1718380)

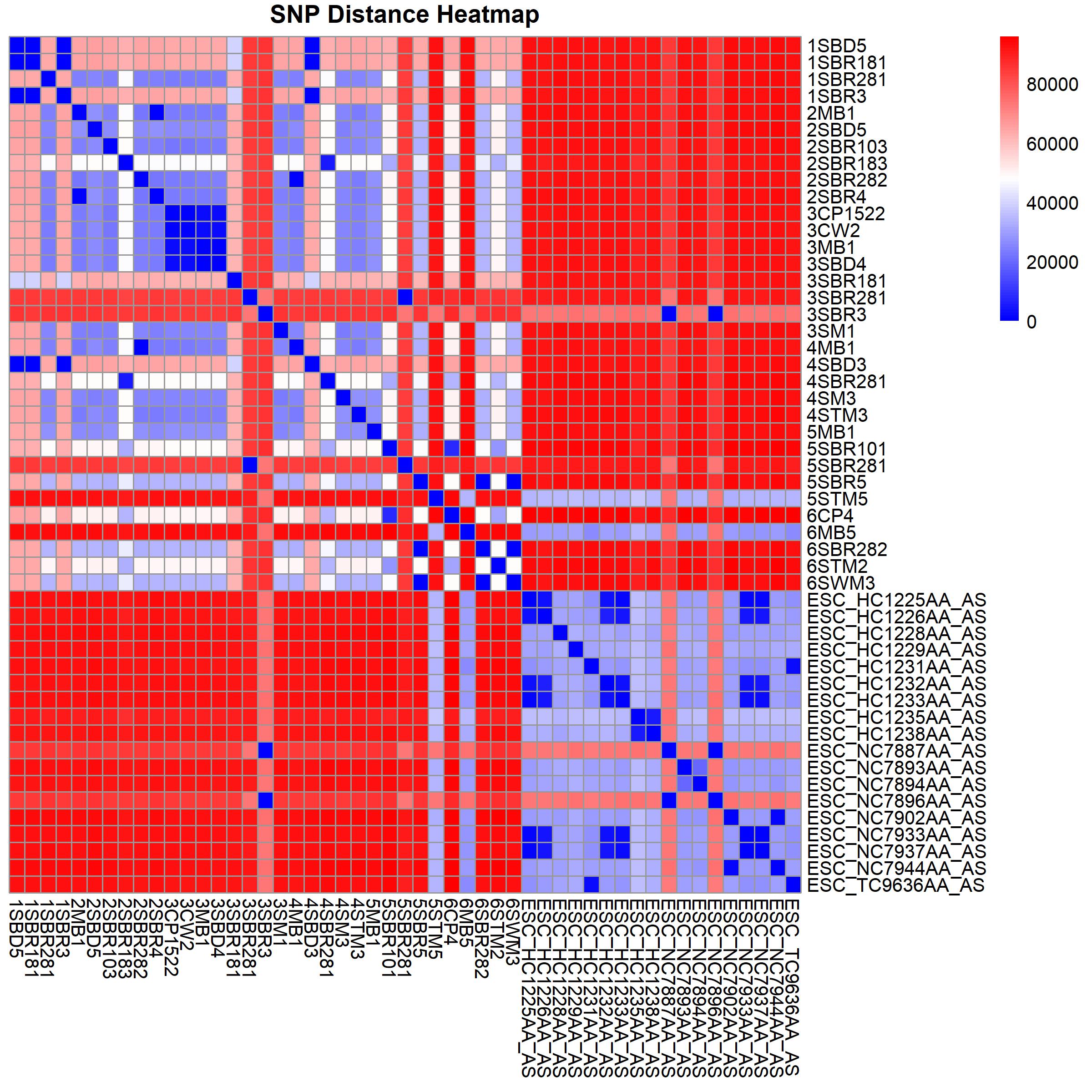

Supplement: SUPPLEMENTARY FIGURE S1 — Heatmap of pairwise SNP distance matrix of the 33 Escherichia coli food genomes and public genomes belonging to phylogenetic clusters ECO1 and ECO2. [file Image_1.JPEG]
